# The Modern and Digital Transformation of Oral Health Care: A Mini Review

**DOI:** 10.3390/healthcare9020118

**Published:** 2021-01-25

**Authors:** Muhammad Syafiq Alauddin, Ahmad Syukran Baharuddin, Mohd Ifwat Mohd Ghazali

**Affiliations:** 1 Department of Conservative Dentistry and Prosthodontics, Faculty of Dentistry, Universiti Sains Islam Malaysia, Kuala Lumpur 56100, Malaysia; 2 Faculty of Syariah and Law, Universiti Sains Islam Malaysia, Nilai 71800, Malaysia; ahmadsyukran@usim.edu.my; 3 Faculty of Science and Technology, Universiti Sains Islam Malaysia, Nilai 71800, Malaysia; ifwat.ghazali@usim.edu.my

**Keywords:** big data, digital dentistry, artificial intelligence, internet of things, augmented reality, dental implants

## Abstract

Dentistry is a part of the field of medicine which is advocated in this digital revolution. The increasing trend in dentistry digitalization has led to the advancement in computer-derived data processing and manufacturing. This progress has been exponentially supported by the Internet of medical things (IoMT), big data and analytical algorithm, internet and communication technologies (ICT) including digital social media, augmented and virtual reality (AR and VR), and artificial intelligence (AI). The interplay between these sophisticated digital aspects has dramatically changed the healthcare and biomedical sectors, especially for dentistry. This myriad of applications of technologies will not only be able to streamline oral health care, facilitate workflow, increase oral health at a fraction of the current conventional cost, relieve dentist and dental auxiliary staff from routine and laborious tasks, but also ignite participatory in personalized oral health care. This narrative article review highlights recent dentistry digitalization encompassing technological advancement, limitations, challenges, and conceptual theoretical modern approaches in oral health prevention and care, particularly in ensuring the quality, efficiency, and strategic dental care in the modern era of dentistry.

## 1. Introduction

Dentistry is a part of the field of medicine that has benefited from the development of modern digital transformation. The utilization of digitalization in dentistry is useful in modern day dentistry especially with numerous challenges involving multiple chronic oral diseases, the complex treatment which is needed by the community with an aging population, and not forgetting continuous rising costs over one’s lifespan [1,2]. The implementation of the digital computer-derived application facilitated by superfast broadband and the internet via smartphones, tablets, personal computers, smart watches is being explored by relevant industries and healthcare providers to deliver comprehensive, yet simplified advanced management in dentistry [2,3]. Computer-generated care with a centralized collection of data has been streamlined, for example in implant dentistry, restorative dentistry, oral and maxillofacial surgery, and others [4,5]. This mini review aims to describe in a brief and concise manner the modern and digital approach used in oral health care by summarizing related literature published on PubMed, Google scholar, and Web of Science. This review is divided into the following discussion topics, namely the utilization of augmented and virtual reality, telemedicine in dentistry particularly in the pandemic era of COVID-19, and additive manufacturing with a focus on implant dentistry, artificial intelligence, digital oral health record, and digital scanners.

## 2. Augmented and Virtual Reality

**Figure 1:**
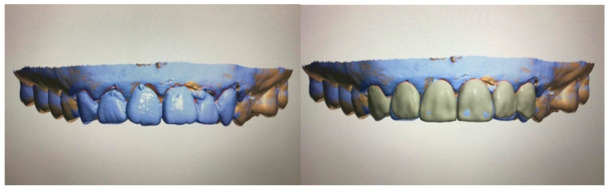
Virtual design in prosthodontics depicting tooth designing done digitally to amend slight rotation and tilting of the front teeth. The left picture shows a misaligned front dentition and the right picture shows the dentition after digital modifications. The final standard triangle language (STL) file is able to be three-dimensionally printed using specific resin via a 3D printer and can be used as a communication tool with the dental technician.

**Figure 2:**
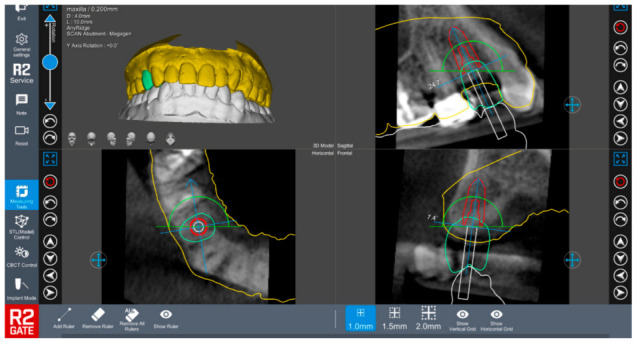
Virtual implant planning utilizing specialized software in which the dental surgeon preplans the surgical implant site using a specialized software to allow precise implant placement prior to the surgery. The figure shows a planned implant placement using R2 Gate™ (R2 Gate, Megagen, South Korea).

Augmented reality (AR) is defined as an interactive development of technology utilizing additional, composite animated information in the user’s real world. AR effectively enhances the user’s real life in the real world through virtual simulation of live imagery and videos. Virtual reality (VR) on the other hand is the total immersion of a user in composite virtual environments in which the user’s feelings, senses, and reactions are virtually simulated by the computer, thus, creating an artificial interaction. Virtual reality systems in the real world normally utilize a device, for example a helmet, with the aim to simulate responses from the user for further exploration based on virtual recreated three and four dimensions [6]. Both systems and techniques are implemented through the user’s sensations either individually or in a combination of all haptic, hearing, auditory, and motor sensations [6,7]. There is a plethora of applications and software which are greatly supported by this technique to enhance the ability and capacity of dental practitioners and dental specialists in providing a total patient care system [6]. In prosthodontics, rehabilitation, reconstruction, and prostheses, designs are augmented to a patient’s true existing anatomy in order to simulate diverse functions and movements without any invasive steps involved (Figure 1) [8]. This allows pre interaction between the dental practitioner and the auxiliary staff, such as a dental technician to prospectively design, and thus decide the best options for a patient’s prosthesis, for example in the construction of dentures utilizing AR/VR systems [9]. On the other hand, oral maxillofacial surgeons, with the aid of specialized software and an engineering team are able to predesign a surgical template or plan based on the current disease manifestation and condition, for example in complex trauma and implant cases (Figure 2) [10]. This allows a more precise surgical technique to be implemented as it reduces the risk of human error in the surgical field as it provides a quicker surgical recovery turnaround, hence, reducing potential unnecessary contamination of the surgical site and surgical morbidity [11,12]. The advancement in AR/VR systems has also enabled better simulation of the three-dimensional digital model, thus, facilitating interaction and communication with the patients [13]. With the recent development of other devices such as intraoral scanner, automated computer-derived manufacturing machine, and modern cone beam computed tomography (CBCT) radiograph machine, it will only grow, facilitate, and rapidly develop the contemporary technique in routine dental care [5,14,15,16,17,18]. Dental education also promises another platform for advancement of the AR/VR system. Theoretical knowledge often delivered based on a traditional classroom environment during the undergraduate dental studies can be further enhanced during the practical skill sessions [19,20]. This does not only allow a receptive feedback environment, but also an interactive teaching space with room for objective and numerical evaluation. The systems will inadvertently improve simulated prospective preclinical training, hand-eye coordination, and ergonomics without the risk of harming a real-life patient [21]. The systems will also allow dental students to reflect and learn by themselves, thus, decreasing the faculty workload as compared to traditional preclinical simulation training. A riskier dental routine procedure such as a dental implant placement protocol and training is better facilitated utilizing the AR/VR technique and environment in order to reduce the potential morbidity associated with it, such as nerve injury, transportation of dental implants to the sinus and wrong implant angulation [11].

## 3. Tele-Dentistry with Remote Consultation

The key to comprehensive oral healthcare is to be based around patient-centered care. The current challenge in this era is the rapid increase of dental treatment cost, the steady incline of population age, the chronic oral diseases that have affected the quality of life, and the need for dental treatment especially for patients from remote areas with difficult-to-reach geographical locations [22]. Telemedicine in dentistry was introduced to facilitate and set a pathway to the patient to reduce the number and timing of dental office visits while at the same time empowering oral health self-care at home. It can be used to aid in diagnosis of caries detection, impacted wisdom teeth detection, screening of oral diseases such as precancerous lesions, and others. This system also provides other advantages, for example in monitoring health conditions and oral health education for elderly patients in assisted communal facilities care. It also bridges the gap for dental care disparities between urban and rural remote communities. It is a powerful additional system to complement the existing technique for delivering oral health care. The treatment objectives have also shifted towards a preventative program rather than the conventional “drill and fill” sequence [23,24]. Remote clinical consultation is a platform that enables sharing of a patient’s data between primary and secondary care as a way to allow a fully integrated comprehensive total patient management system by using a superfast internet connection utilizing visual and audio aid streaming [25]. This will enable simultaneous discussion and decision to occur among patient, dentist, and specialist, thus, enabling a comprehensive oral health care to take place. This system will prevent unnecessary travelling and allow the review or consultation to be conducted at home, at communal facilities, or primary care settings. It will effectively prevent and minimize the risk of infection, especially to the immunocompromised community like the elderly, people with chronic disease such as asthma, heart disease, renal failure, and children [26,27,28,29,30].

## 4. Additive Manufacturing

Additive manufacturing is a rapid production process of any three-dimensional (3D) object using 3D printers. The process allows a complex geometrical design to be produced with additional benefits such as a reduction of unnecessary raw material wastage, mass production of the desired items, and fast production and manufacturing of dental prostheses as compared to subtractive manufacturing [31]. In dentistry, additive manufacturing has been adopted in multiple dental fields such as prosthodontics, implant dentistry, oral surgery, and others [32,33]. The production of a chairside dental model through a 3D printing method allows a quick reference to the dentist after the virtual designing is completed, thus, facilitating the treatment plan and communication between the dentist and patient [34]. A surgical guide for implant placement which is produced by the additive manufacturing method will allow the precise placement of a dental implant. This technique will help to eliminate a possible complication in which the vital nerve and blood vessels are traumatized. It will instead permit a “prosthesis-driven implant placement”. Moreover, a surgical guide is a specifically designed tool which utilizes multiple specialized softwares to adopt the virtual simulation process prior to the surgical appointment [35,36]. Due to the limitation of subtractive manufacturing such as the milling process, 3D printing is considered as the solution from a scientific and technical point of view. The overall schematic flow of the utilization of an additive manufacturing is simplified in Figure 2 with the example emphasized in implant surgical guide construction and fabrication protocol. Owing to the rapid development and thriving evolution of the implant rehabilitation utilizing additive manufacturing, the author briefly discusses the contemporary update on the field particularly on the surgical static and dynamic system.

### 4.1. Additive Manufacturing for Surgical Guide in Implant Rehabilitation

The conventional implant rehabilitation and therapy usually utilizes multiple gypsum casts for medical record and references, in addition to two-dimensional radiographical imaging (periapical and orthopantomogram view) and chairside clinical examination. The inherent disadvantages of this method include far less accuracy in interpretation of the vital anatomical and osseous structures as compared to cone beam computed tomography (CBCT), limited gypsum material properties, potential error-prone manual laboratory procedural, and others [37]. The development and rapid usage of CBCT imaging in implant therapy has allowed for a more specific and detailed implant site with marked decrease in exposure to radiation. Initially, the adoption of CBCT in implant therapy is to serve the diagnostic purpose of evaluating osseous resorption pattern, identifying of vital anatomical structures, and examining the residual alveolar bone [38]. Then, the further progress in modern technologies led to the development of a virtual three-dimensional implant planning software by implant manufacturers as a means to allow a fabrication of implant treatments with the concept of prosthesis-driven implant therapy (Table 1). With this, dental implants may be placed almost anywhere as long as the osseous condition permits, but correct fundamental planning must be done beforehand to ensure the final implant prosthesis fulfills the acceptable aesthetic profile. This specialized software will then link adherently to the interpretation of the anatomical structures which are derived from the CBCT, virtual planning of surgical and prosthesis, and accurate surgical and prostheses intervention. This provides numerous advantages to dental practitioners including previsualizing and premeasurement of important anatomical landmark and structures, accurate implant placement to satisfy both functional and aesthetic profile, profiling the final implant prosthesis at the earliest planning stage, predictable surgical stage with less clinical stress to the practitioners, reducing significant amount of chairside time, and the ability to learn a case difficulty and challenges ahead of time [39,40,41]. That information is briefly described in Table 2 together with the commercial global brand of manufacturers accompanying the surgical guide material in Table 3. This development promotes the implementation of technology in a computer-aided surgery (CAS) implant placement conceptual system. This conceptual protocol has thus far been used extensively in the field of medicine, particularly orthopedic surgery and neurosurgery [42]. The concepts can be further divided into computer-guided (static) and computer-navigated (dynamic) systems.

**Figure 3:**
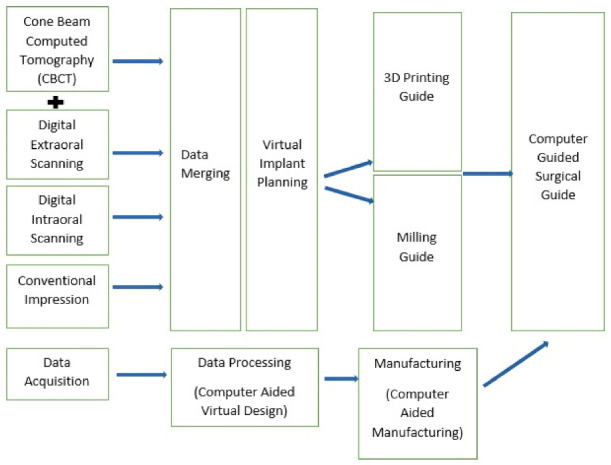
Schematic workflow of static guided surgery. Please note the 3D printing in the manufacturing stage.

The conventional digital workflow on computer guided surgical guide is as given in the schematic diagram below (Figure 3).

#### 4.1.1. Static Guided Systems

**Figure 4:**
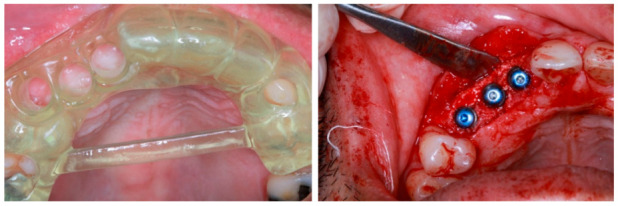
The example of a surgical implant guide (left) virtually designed via a specialized computer software to allow a precise and accurate implant placement with the desirable inter implant distance as depicted on the right picture. The surgical guide is produced by utilizing an additive manufacturing method manufactured by a 3D printer.

The static guided system is a computer-guided derived three-dimensional virtual implant planning that transfers the information into a pre-planned fabricated surgical template. It can be divided into two major types, which are the fully guided implant surgery utilizing full specialized osteotomy kit on guided prefabricated surgical template and partial-guided that utilizes analogue fabrication (non-computer guided) which is pilot-guided and drilling-guided [43]. Another classification of the static surgical guided is further classified into four categories; tooth-supported, mucosa-supported, bone-supported, and specialized implants or pins-supported guided template [43,44]. There is a consensus in the literature which emphasizes the fact that tooth- and mucosa-supported is not only the most stable, but also has the highest accuracy as compared to mucosa- and bone-supported. Full surgical-guided is viewed as being better than partial-guided and non-guided. On the other hand, flapless surgery is much better than flap in partial edentulous cases, whereas, flap surgery is considered a better choice in comparison to a flapless surgery in full edentulous as it has a much higher safety margin needed for full edentulous cases [45]. An example of clinical application of static surgical guide are photographed as in Figure 4.

Stereolithography (SLA) is the most common additive manufacturing technology in fabrication of static surgical guide utilizing computer-aided design and computer-aided manufacturing (CAD-CAM) protocol with specialized resin required as briefly listed in Table 3. The surgical guide will be fabricated first in the early stage, then followed by the placement of metal sleeve from manufacturers according to the implant size, diameter, length and depth of placement. Most implant manufacturers require a drill guide and/or a specialized guided surgical osteotomy kit. There are only a few implant manufacturers who promote surgical guides without the need to use a drill guide and the metal sleeve (R2 Navi Guide™, Megagen, Daegu, South Korea), which empirically is proven to be clinically reliable [46,47].

#### 4.1.2. Dynamic Navigation System (DNS)

The CAS method is classified as dynamic when real-time computer-assisted programs and tracking devices are used to guide the practitioners placing the implant into the pre-planned insertion pathways intraoperatively [48]. The main characteristic of this dynamic navigation system (DNS) is that it allows the practitioner to visualize the implant site on the computer screen during the osteotomy and implant placement protocol. The practitioners are able to modify and amend, if necessary, the plan of the implant surgery during the day of the surgery [48,49]. The list of contemporary DNS available in the market are in Table 4. Several beneficial characteristics in DNS include the implementation of a one-day implant therapy protocol which encompasses scanning, planning, and surgical protocol on the same day. In addition to being flexible and having a versatile planning system, it can also visualize the surgery and have the ability to verify the accuracy at all times. It is potentially also able to reduce the number of visits for the whole implant therapy procedure with user- and clinician-friendly software and setup [50,51]. In the perspective of practicality and from the standpoint of a learning curve, two studies clearly showed that there was no significant difference between experienced and novice professionals when comparing between DNS and free hand placement as novice professionals are able to improve tremendously after multiple attempts on DNS [52,53]. Nevertheless, DNS requires long planning and chairside time. It also requires a high initial economical spending and regular calibration of drills and implants. There is also the risk of inaccuracy during the registration stage, in addition to having a steep initial learning curve.

## 5. Artificial Intelligence (AI)

Artificial Intelligence (AI) is the ability of a machine to perform human tasks. It revolves around the ability of a machine, around its own intelligence, to solve problems based on the learning of a specific set of data. The foundation of AI is to increase the ability of machines or its intelligence components to perform tasks with speed, low resources, accuracy, and others [54,55]. It will also eliminate human intervention such as potential human error, emotion, and bias, thus, making it a perfect solution for laborious work with increased risk of error. Other potential human symptoms such as fatigue, tiredness, and boredom after a continuous repetitive work are also eliminated [4]. It cannot be emphasized enough that AI requires advanced machine learning on huge datasets (“big data”) as it utilizes specific algorithm to perform the required works [56].

The application of AI in dentistry is huge with enormous potential [56]. In dental radiology, the application of AI by using cone beam computed tomography allows an automated detection of specific landmarks such as in lateral cephalometric view, dental panoramic radiography, and others. The progress is also encouraging through other techniques such as automated detection of caries, periodontal disease, periapical disease, and detection of possible oral disease such as cysts or tumor [57]. In restorative dentistry, AI has been developed to suggest and detect the presence of dental caries [57]. The conventional method of the detection of caries includes a combination of visual tactile examinations with special investigations such as dental bitewing radiograph. Nonetheless, the evolution in AI challenges the normal paradigm in caries detection with the utilization of state of the art, multiple varieties of neural networks including convoluted neural network, artificial neural networks, and additional derivatives of neural network architecture such as ResNet 18 and ResNext50 [58]. A systematic review by Pravos-Privado et al. showed that despite the advancement of AI systems in caries detection, various heterogeneity aspects such as parameters, multiple neural network systems, and outcome complicates the accuracy, diagnostics performance, specificity, and sensitivity of AI in caries detection [59]. Another study by Hung et al. proved that machine learning can be utilized for root caries prediction and prognostic value on a large mass of population data using numerous demographics, general and oral health, social, and lifestyle variables [60]. Another machine learning algorithm, support vector machine (SVM) showed high predictability and accuracy in assessing the level of complexity of root canal treatments based on American Association of Endodontics Case Difficulty Assessment Forms and thus, are able to aid the classification, clinical cognitive decision by the clinician, and the overall rapid referral process done by general dental practitioners [61]. In an endodontics diagnostics procedure, the advancement of AI was utilized to detect the periapical pathology by using a prototype of deep convolutional neural network through mass data from CBCT with a high reliability in comparison with the manual conventional segmentation method [62]. This is due to the ability of deep learning and convoluted neural network of AI components to segment and integrate the original data from radiographic CBCT views and images which then further coded these formats into a form that can be evaluated later on. The advanced development of computer aided design and computer aided manufacturing also allows software to precisely construct dental prostheses despite complex geometrical shapes and laborious laboratory workflow with the potential of a high risk of error involved [63]. The dental restoration must be a perfect fit, able to undergo ideal function, and also be aesthetically pleasing. In orthodontics-driven AI, a diagnosis, monitoring, and a specific yet individualized treatment plan is available. The clear aligners are produced based on accurate 3D model scanning and dental models. The AI creates an algorithm in which it can predict and decide future tooth movement, and the necessary pressure to be applied to the teeth with aid and input from the dental practitioner [64]. AI is designed to facilitate the construction of a surgical guide in specialized software with its major role in detecting the thickness, the height, and the density of the bone based on data acquisition from cone beam computed tomography [11]. This will facilitate the dental practitioner’s decision on suitable timing and technique for the implant placement. Diagnosis of temporomandibular joint disorders (TMDs) can be detected based primarily on history and signs and symptoms, which are then to be followed by clinical examinations. Theoretically, the collective clinical data of TMDs examinations, if they can be translated into a well-structured and organized computer language, are able to differentiate between absolute TMDs diagnosis and other clinical conditions mimicking TMDs [65]. A study by Shoukri et al. (2019) showed that the neural networks are able to program and classify the TMDs based on the combination of condylar radiographic imaging utilizing CBCT, biological markers such as saliva, and a variable range of clinical indicators including detailed facial and muscle pain and soreness history, range of mouth opening, and other signs such as headaches [66].

## 6. Ethical Issues and Challenges in AR/VR and AI

The application of AR/VR in healthcare services, particularly in dentistry, may give rise to issues such as enormous, massive data availability and trusted sharing. The privacy of a patient’s data is handled by a series of systems utilizing software type algorithms intended to represent human cognitive processes in clinical decision-making. The dental practitioner and the auxiliary teams are responsible for data handling with potential risks of data privacy and security breaches. The AI/VR systems are not held accountable for this, though the application is performed either under supervision or not within the legal and jurisprudence context [65,67].

The emerging wealth disparities among communities widens each year further representing the economic distribution and inequalities. Theoretically, the utilization of AR/VR and AI will streamline the workforce and reduce laborious, repetitive tasks and the need for additional manpower. In the long run, it is predominantly an effective way to reduce the operating cost and increase the revenues for healthcare industries. Nevertheless, the shift of physical, repetitive jobs to a more complex, cognitive driven one as required in the globalized society and industries will effectively reduce the need for human involvement in low-scale, laborious tasks causing depletion of available jobs in the healthcare sector. Hence, there will be issues in a fair, post-labor economy with division of an income-based society in the future [68].

The infrastructure support like computing power and requirements are critical to ensure the smooth processing of data updating, gathering, and interpretation in the delivery of the oral health care system which utilizes AI. The continuous expanding data of patients including demographic, clinical, treatment, and follow up requires consistent upgrading of the computing power which potentially may give rise to health, economic, training, technical logistics, and maintenance issues [65,69]. The inability to match the expected computational resources will reduce the efficacy of the AI, thus, reducing the delivery of the modern healthcare services. With that in mind, the common ideal solution for this is to utilize quantum supercomputing which can process the conventional binary bit using quantum version (qubits) which is fundamentally faster than conventional computing systems [70,71].

## 7. Digital Oral Health Records

The form of population-derived health records linked at the personalized individual level of information and data is a great model for the health economic policy. The health data can be derived from conventional medical and dental screening, routine check-ups, follow-ups, and hospitalization together with other determinants such as socio-economic (income, jobs) and other social aspects (housing, food, security) [56]. Additionally, any other public information such as participation of the patient in online surveys, research, and forums in the context of Internet of medical things (IoMT) can be further evaluated and gathered [56]. This can be facilitated by the exponential usage of digital devices including smartphone, laptop, smartwatches, and digital online application facilitated by a superfast internet connection. Mass digital dental data and records are advantageous as they can be utilized by stakeholders for analytical disease prediction and prognostics modeling, population preventative programs, clinical research and surveys, clinical support systems, association of factors and cofounding factors for disease, identification of novel disease and treatment concepts, and the overall governance and delivery of the oral healthcare system. These population-based, yet personalized electronic digital oral health records will be an excellent platform and medium for interconnection between the dentist, dental auxillary team such as dental therapist and dental nurses, specialists, and physicians to understand, collaborate, and deliver an optimum level of oral healthcare through an interdisciplinary manner [56,72,73].

## 8. Digital Oral Scanner

The conventional impression has played a part in the prosthodontics field in which the main aim of the procedure is to replicate and simulate the functional anatomical parts intraorally. Nevertheless, the challenge lies in producing it due to material disadvantages, namely volumetric shrinkage and expansion of dental stone and silicones, technique sensitivity and critical handling having to be done by the clinician, and others. This procedure is thus prone to error and inadvertently will affect the final prostheses outcome [74,75,76].

The current development of computer-aided design and computer-aided manufacturing (CAD-CAM) has brought the prosthodontics field to a new frontier. The construction of dental prostheses has been rapidly evolving with the influence of subtractive manufacturing with the example of machinable milling systems and additive manufacturing in 3D printers that have resulted in rapid prosthesis production and shortened manufacturing time. Three-dimensional (3D) dental models are obtained by intraoral scanners, thus, eliminating the need for the conventional impression method. The advantages include less time required for the impression taking procedure, avoiding cross-contamination risks, better communication tools for dental technicians and patients, as well as more simplified procedures for the clinician [76,77].

Dental prostheses are made available through direct fabrication using a CAD-CAM system after obtaining a digital impression with an intraoral scanner. This will eliminate the need for physical models. However, manual fabrication and the construction of dental prostheses are still required in the construction of lithium disilicate restoration in which manual veneering wax-up must be done and the casting of metal alloy for full or partial coverage for gold restoration [78]. An extraoral laboratory scanner then scans the dental impression or gypsum casts to create a 3D model, and the restoration is then designed on computer with a specialized design software and then 3D printed. This protocol is beneficial to the dental technician and even useful for clinicians who are still using the conventional impression method but require prostheses that adopt the CAD-CAM approach with the example of monolithic zirconia restoration [79].

It is paramount to evaluate the accuracy of these digital approaches, especially the intraoral scanner since it is the first step in adopting the CAD-CAM approach. Multiple studies had evaluated the accuracy of the intraoral scanner; however, they were limited to the production of orthodontic appliances such as retainers and implant prosthodontics [80,81,82,83]. As defined by International Organization for Standardization (ISO-5725), accuracy consists of trueness and precision. Trueness refers to the closeness of the experimental result to the true value. High trueness indicates that the experimental result is very close or equivalent to the true value. In contrast, precision describes the closeness of agreement between intragroup data obtained by repetitive measurements [84]. A number of studies had evaluated the accuracy between intraoral scanners for fixed dental prosthesis; nevertheless, the results are debatable. Zimmermann et al. reported that each intraoral scanner with a different CAD system has a different value of accuracy [85]. Kim et al. reported that CS3600 by Carestream Dental USA and conventional impression with laboratory scanning had the highest accuracy in production of zirconia crown; however, this study does not include other relevant, commercially available intraoral and extraoral scanners [86]. Nedelcu et al. showed that in the production of a distinctive finish line in full coverage crown scanning, CareStream 3600 and Trios 3 by 3 Shape showed better accuracy although this study focused on finishing line integrity [87]. Meanwhile, Planmeca Planscan intraoral scanning devices showed the least accurate digital impression by Mennito et al. [88]. Another elaborative study by Mangano et al., in full arch implant impression, comparing the trueness of 12 different intraoral scanners utilizing multiple variables outcomes including mesh/mesh evaluation and nurbs/nurbs evaluation showed that there was a significant difference in terms of accuracy between different intraoral scanners that are commercially available worldwide [89].

## 9. Conclusions

The delivery of modern oral healthcare should be derived based on modern technology driven by a patient-centered outcome. Digitalization in dentistry will facilitate oral healthcare to an optimum level. The pandemic of COVID-19 showed that tele-dentistry with remote consultation and artificial intelligence has a major role to play. It will indefinitely reduce the unnecessary contact between the patients and healthcare providers, shorten the duration of treatment, and be more cost effective in the long run. The field of dentistry is most likely to benefit especially in the utilization of AR/VR and AI systems for the delivery of pedagogy and clinical skills teaching. The research on digitalization in healthcare especially in dentistry should be the main focus in the next few decades with the aim of improving data acquisition and big datasets, safety and security of the “Big Data”, updating the neural networks, machined and deep learning of artificial intelligence, and other relevant fields.

## Figures and Tables

**Table 1:** Example of a static guided implant planning system in the market.

Manufacturer	Software
3 Shape	Implant Studio
Nobel Biocare	NobelClinician
Straumann	CoDiagnostix™
Sirona	SICAT
Materialise	SimPlant^®^
Bredent	SKYplanX
360Imaging	360dps
BlueSky bio	BlueskyPlan
Anatomage	Anatomage guide
AstraTech dental	Facilitate
BioHorizons	VIP 3
CyberMed	OnDemand3D™
Swissmeda AG	Swissmeda Planning Solution
SICAT GmbH & Co. KG	SICAT Implant 2.0
MIS	MGUIDE
Megagen Implant	R2 Gate
OSSTEM	OneGuide
Exocad	Implant Module
Amann Girrbach	Ceramill M-Plant (abutment module only)
Planmeca	Planmeca Romexis^®^ 3D

**Table 2:** List of advantages and disadvantages of implementing surgical static guided surgery.

Advantages	Disadvantages
Avoid risks of injuring important anatomical structures	Steep initial learning curve
Involve multidisciplinary approaches	High initial cost
Possible avoidance of complex bone regeneration/grafting technique	Increased preoperative surgical planning
Reduced surgical chairside time	Adequate mouth opening; challenges for microstomia patient or posterior implant placement.
Allow minimal surgical intervention (flapless surgery)	Limited visual on implant crestal depth location
Improve dentist-patient communication due to required preoperative planning	Risk of fracture on the surgical templates

**Table 3:** List of three-dimensional (3D) surgical guide materials (resin) from manufacturers.

Manufacturer	Material
NextDent	NexDent-SG
Stratasys	MED610
EnvisionTec	E-Guide Tint
Formlabs	Surgical Guide Resin Dental SG Resin
Zortrax	Raydent Surgical Guide Resin
BEGO	VarseoWax Surgical Guide
SHERA	SHERAprint-sg
DentalMed	3Delta Guide S
Carbon	Whip Mix Surgical Guide
Detax	FREEPRINT^®^ splint 2.0
3D Systems	Visijet M3 Stoneplast
Zenith	ZMD-1000B CLEAR-SG
SprintRay	SprintRay Surgical Guide 2
Shining 3D^®^	Resin Shining 3D Surgical Guide
Prodways Tech	PLASTCure Clear 200
DMG	LuxaPrint Ortho
UNIZ	zSG (Surgical Guide) Resin
3Dresyns	Dental 3Dresyns OD
Makex	Surgical Guide
VOCO	V-Print SG

**Table 4:** Example of dynamic navigation implant systems available in the market.

Manufacturer	System
ClaroNav Technology Inc.	Navident
X-Nav Technologies	X Guide™
Image Navigation	Image Guided Implant (IGI) Dentistry System
Neocis	YOMI^®^
Navigate Surgical	Inliant^®^

## References

[1] Thomson W.M., Ma S. (2014). An ageing population poses dental challenges. Singap. Dent. J..

[2] Favaretto M., Shaw D., De Clercq E., Joda T., Elger B.S. (2020). Big Data and Digitalization in Dentistry: A Systematic Review of the Ethical Issues. Int. J. Environ. Res. Public Health.

[3] Nilsen W., Kumar S., Shar A., Varoquiers C., Wiley T., Riley W.T., Atienza A.A. (2012). Advancing the science of mHealth. J. Health Commun..

[4] Joda T., Bornstein M.M., Jung R.E., Ferrari M., Waltimo T., Zitzmann N.U. (2020). Recent trends and future direction of dental research in the digital era. Int. J. Environ. Res. Public Health.

[5] Rekow E.D. (2020). Digital dentistry: The new state of the art—Is it disruptive or destructive?. Dent. Mater..

[6] Huang T.K., Yang C.H., Hsieh Y.H., Wang J.C., Hung C.C. (2018). Augmented reality (AR) and virtual reality (VR) applied in dentistry. Kaohsiung J. Med. Sci..

[7] Farronato M., Maspero C., Lanteri V., Fama A., Ferrati F., Pettenuzzo A., Farronato D. (2019). Current state of the art in the use of augmented reality in dentistry: A systematic review of the literature. BMC Oral Health.

[8] Ogawa T., Ikawa T., Shigeta Y., Kasama S., Ando E., Fukushima S., Suzuki N. Virtual reality image applications for treatment planning in prosthodontic dentistry. Proceedings of the MMVR 2011.

[9] Raja’a M., Farid F. (2016). Computer-based technologies in dentistry: Types and applications. J. Dent..

[10] Ayoub A., Pulijala Y. (2019). The application of virtual reality and augmented reality in Oral & Maxillofacial Surgery. BMC Oral Health.

[11] Ferro A.S., Nicholson K., Koka S. (2019). Innovative Trends in Implant Dentistry Training and Education: A Narrative Review. J. Clin. Med..

[12] Pellegrino G., Mangano C., Mangano R., Ferri A., Taraschi V., Marchetti C. (2019). Augmented reality for dental implantology: A pilot clinical report of two cases. BMC Oral Health.

[13] Chander N.G. (2019). Augmented reality in prosthodontics. J. Indian Prosthodont. Soc..

[14] Maspero C., Farronato M., Bellincioni F., Annibale A., Machetti J., Abate A., Cavagnetto D. (2020). Three-dimensional evaluation of maxillary sinus changes in growing subjects: A retrospective cross-sectional study. Materials.

[15] Lanteri V., Farronato M., Ugolini A., Cossellu G., Gaffuri F., Parisi F.M., Cavagnetto D., Abate A., Maspero C. (2020). Volumetric Changes in the Upper Airways after Rapid and Slow Maxillary Expansion in Growing Patients: A Case-Control Study. Materials.

[16] Maspero C., Farronato M., Bellincioni F., Cavagnetto D., Abate A. (2020). Assessing mandibular body changes in growing subjects: A comparison of CBCT and reconstructed lateral cephalogram measurements. Sci. Rep..

[17] Farronato M., Cavagnetto D., Abate A., Cressoni P., Fama A., Maspero C. (2020). Assessment of condylar volume and ramus height in JIA patients with unilateral and bilateral TMJ involvement: Retrospective case-control study. Clin. Oral Investig..

[18] Maspero C., Abate A., Bellincioni F., Cavagnetto D., Lanteri V., Costa A., Farronato M. (2019). Comparison of a tridimensional cephalometric analysis performed on 3T-MRI compared with CBCT: A pilot study in adults. Prog. Orthod..

[19] Roy E., Bakr M.M., George R. (2017). The need for virtual reality simulators in dental education: A review. Saudi Dent. J..

[20] Othman N.I., Ismail H.U., Mohammad N., Ghazali N., Alauddin M.S. (2020). An Evaluation on Deep Caries Removal Method and Management Performed by Undergraduate Dental Students: A Malaysia Experience. Eur. J. Dent..

[21] Towers A., Field J., Stokes C., Maddock S., Martin N. (2019). A scoping review of the use and application of virtual reality in pre-clinical dental education. Br. Dent. J..

[22] Besimo C.E., Zitzmann N.U., Joda T. (2020). Digital Oral Medicine for the Elderly. Int. J. Environ. Res. Public Health.

[23] Khan S.A., Omar H. (2013). Teledentistry in practice: Literature review. Telemed. E-Health.

[24] Jampani N.D., Nutalapati R., Dontula B.S.K., Boyapati R. (2011). Applications of teledentistry: A literature review and update. J. Int. Soc. Prev. Community Dent..

[25] Martin N., Shahrbaf S., Towers A., Stokes C., Storey C. (2020). Remote clinical consultations in restorative dentistry: A clinical service evaluation study. Br. Dent. J..

[26] Yadav V., Kumar V., Sharma S., Chawla A., Logani A. (2020). Palliative dental care: Ignored dimension of dentistry amidst COVID-19 pandemic. Spec. Care Dent..

[27] Crawford E., Taylor N. (2020). The effective use of an e-dentistry service during the COVID-19 crisis. J. Orthod..

[28] Santana L.A.D.M., Santos M.A.L.D., Albuquerque H.I.M.D., Costa S.F.D.S., Rezende-Silva E., Gercina A.C., Takeshita W.M. (2020). Teledentistry in Brazil: A Viable Alternative during COVID-19 Pandemic. Rev. Bras. Epidemiol..

[29] Talla P.K., Levin L., Glogauer M., Cable C., Allison P.J. (2020). Delivering dental care as we emerge from the initial phase of the COVID-19 pandemic: Teledentistry and face-to-face consultations in a new clinical world. Quintessence Int..

[30] Maspero C., Abate A., Cavagnetto D., El Morsi M., Fama A., Farronato M. (2020). Available technologies, applications and benefits of teleorthodontics. A literature review and possible applications during the COVID-19 Pandemic. J. Clin. Med..

[31] Tofail S.A., Koumoulos E.P., Bandyopadhyay A., Bose S., O’Donoghue L., Charitidis C. (2018). Additive manufacturing: Scientific and technological challenges, market uptake and opportunities. Mater. Today.

[32] Dawood A., Marti B.M., Sauret-Jackson V., Darwood A. (2015). 3D printing in dentistry. Br. Dent. J..

[33] Kessler A., Hickel R., Reymus M. (2020). 3D printing in dentistry—state of the art. Oper. Dent..

[34] Bukhari S., Goodacre B.J., AlHelal A., Kattadiyil M.T., Richardson P.M. (2018). Three-dimensional printing in contemporary fixed prosthodontics: A technique article. J. Prosthet. Dent..

[35] Ma B., Park T., Chun I., Yun K. (2018). The accuracy of a 3D printing surgical guide determined by CBCT and model analysis. J. Adv. Prosthodont..

[36] Yeung M., Abdulmajeed A., Carrico C.K., Deeb G.R., Bencharit S. (2020). Accuracy and precision of 3D-printed implant surgical guides with different implant systems: An in vitro study. J. Prosthet. Dent..

[37] Unsal G.S., Turkyilmaz I., Lakhia S. (2020). Advantages and limitations of implant surgery with CAD/CAM surgical guides: A literature review. J. Clin. Exp. Dent..

[38] Greenberg A.M. (2015). Digital technologies for dental implant treatment planning and guided surgery. Oral. Maxillofac. Surg. Clin..

[39] Joda T., Ferrari M., Gallucci G.O., Wittneben J.G., Brägger U. (2017). Digital technology in fixed implant prosthodontics. Periodontology 2000.

[40] Joda T., Zarone F., Ferrari M. (2017). The complete digital workflow in fixed prosthodontics: A systematic review. BMC Oral Health.

[41] Colombo M., Mangano C., Mijiritsky E., Krebs M., Hauschild U., Fortin T. (2017). Clinical applications and effectiveness of guided implant surgery: A critical review based on randomized controlled trials. BMC Oral Health.

[42] Joskowicz L. (2017). Computer-aided surgery meets predictive, preventive, and personalized medicine. EPMA J..

[43] Tatakis D.N., Chien H.H., Parashis A.O. (2019). Guided implant surgery risks and their prevention. Periodontol. 2000.

[44] D’Souza K.M., Aras M.A. (2012). Types of implant surgical guides in dentistry: A review. J. Oral Implantol..

[45] Gargallo-Albiol J., Barootchi S., Salomó-Coll O., Wang H.L. (2019). Advantages and disadvantages of implant navigation surgery. A systematic review. Ann. Anat. Anat. Anz..

[46] Mouhyi J., Salama M.A., Mangano F.G., Mangano C., Margiani B., Admakin O. (2019). A novel guided surgery system with a sleeveless open frame structure: A retrospective clinical study on 38 partially edentulous patients with 1 year of follow-up. BMC Oral Health.

[47] Tallarico M., Meloni S.M., Martinolli M., Xhanari E. (2019). Accuracy of sleeveless surgical templates-one-year randomized controlled trial. Clin. Oral Implant. Res..

[48] Emery R.W., Merritt S.A., Lank K., Gibbs J.D. (2016). Accuracy of dynamic navigation for dental implant placement–model-based evaluation. J. Oral Implantol..

[49] Mandelaris G.A., Stefanelli L.V., DeGroot B.S. (2018). Dynamic navigation for surgical implant placement: Overview of technology, key concepts, and a case report. Compend. Contin. Educ. Dent..

[50] Block M.S., Emery R.W. (2016). Static or dynamic navigation for implant placement—choosing the method of guidance. J. Oral Maxillofac. Surg..

[51] Block M.S., Emery R.W., Cullum D.R., Sheikh A. (2017). Implant placement is more accurate using dynamic navigation. J. Oral Maxillofac. Surg..

[52] Jorba-García A., Figueiredo R., González-Barnadas A., Camps-Font O., Valmaseda-Castellón E. (2019). Accuracy and the role of experience in dynamic computer guided dental implant surgery: An in-vitro study. Med. Oralpatologia Oral Y Cir. Bucal.

[53] Golob Deeb J., Bencharit S., Carrico C.K., Lukic M., Hawkins D., Rener-Sitar K., Deeb G.R. (2019). Exploring training dental implant placement using computer-guided implant navigation system for predoctoral students: A pilot study. Eur. J. Dent. Educ..

[54] Currie G. (2019). Intelligent imaging: Anatomy of machine learning and deep learning. J. Nucl. Med. Technol..

[55] Park W.J., Park J.B. (2018). History and application of artificial neural networks in dentistry. Eur. J. Dent..

[56] Joda T., Waltimo T., Pauli-Magnus C., Probst-Hensch N., Zitzmann N.U. (2018). Population-based linkage of big data in dental research. Int. J. Environ. Res. Public Health.

[57] Hung K., Yeung AW K., Tanaka R., Bornstein M.M. (2020). Current Applications, Opportunities, and Limitations of AI for 3D Imaging in Dental Research and Practice. Int. J. Environ. Res. Public Health.

[58] Schwendicke F., Elhennawy K., Paris S., Friebertshäuser P., Krois J. (2020). Deep learning for caries lesion detection in near-infrared light transillumination images: A pilot study. J. Dent..

[59] Prados-Privado M., García Villalón J., Martínez-Martínez C.H., Ivorra C., Prados-Frutos J.C. (2020). Dental Caries Diagnosis and Detection Using Neural Networks: A Systematic Review. J. Clin. Med..

[60] Hung M., Voss M.W., Rosales M.N., Li W., Su W., Xu J., Bounsanga J., Ruiz-Negrón B., Lauren E., Licari F.W. (2019). Application of machine learning for diagnostic prediction of root caries. Gerodontology.

[61] Mallishery S., Chhatpar P., Banga K.S., Shah T., Gupta P. (2019). The precision of case difficulty and referral decisions: An innovative automated approach. Clin. Oral Investig..

[62] Orhan K., Bayrakdar I.S., Ezhov M., Kravtsov A., Özyürek T.A. (2020). Evaluation of artificial intelligence for detecting periapical pathosis on cone-beam computed tomography scans. Int. Endod. J..

[63] Takahashi T., Nozaki K., Gonda T., Ikebe K. (2020). A system for designing removable partial dentures using artificial intelligence. Part 1. Classification of partially edentulous arches using a convolutional neural network. J. Prosthodont. Res..

[64] Kunz F., Stellzig-Eisenhauer A., Zeman F., Boldt J. (2020). Artificial intelligence in orthodontics. J. Orofac. Orthop. Fortschr. Der Kieferorthopädie.

[65] Shan T., Tay F.R., Gu L. (2020). Application of Artificial Intelligence in Dentistry. J. Dent. Res..

[66] Shoukri B., Prieto J.C., Ruellas A., Yatabe M., Sugai J., Styner M., Zhu H., Huang C., Paniagua B., Aronovich S. (2019). Minimally invasive approach for diagnosing TMJ osteoarthritis. J. Dent. Res..

[67] Currie G., Hawk K.E., Rohren E.M. (2020). Ethical principles for the application of artificial intelligence (AI) in nuclear medicine. Eur. J. Nucl. Med. Mol. Imaging..

[68] Fiske A., Henningsen P., Buyx A. (2019). Your robot therapist will see you now: Ethical implications of embodied artificial intelligence in psychiatry, psychology, and psychotherapy. J. Med. Internet Res..

[69] Sunny S., Baby A., James B.L., Balaji D., Rana M.H., Gurpur P., Skandarajah A., D’Ambrosio M., Ramanjinappa R.D., Mohan S.P. (2019). A smart tele-cytology point-of-care platform for oral cancer screening. PLoS ONE.

[70] Hopper H., Ranjan M. (2019). What If Quantum Computer Combined with Artificial Intelligence?. Sci. Insigt..

[71] Sarma S.D., Deng D.L., Duan L.M. (2019). Machine learning meets quantum physics. arXiv.

[72] Nanayakkara S., Zhou X., Spallek H. (2019). Impact of big data on oral health outcomes. Oral Dis..

[73] Di Sanzo M., Cipolloni L., Borro M., La Russa R., Santurro A., Scopetti M., Simmaco M., Frati P. (2017). Clinical applications of personalized medicine: A new paradigm and challenge. Curr. Pharm. Biotechnol..

[74] Schaefer O., Schmidt M., Goebel R., Kuepper H. (2012). Qualitative and quantitative three-dimensional accuracy of a single tooth captured by elastomeric impression materials: An in vitro study. J. Prosthet. Dent..

[75] Soganci G., Cinar D., Caglar A., Yagiz A. (2018). 3D evaluation of the effect of disinfectants on dimensional accuracy and stability of two elastomeric impression materials. Dent. Mater. J..

[76] Mangano F., Gandolfi A., Luongo G., Logozzo S. (2017). Intraoral scanners in dentistry: A review of the current literature. BMC Oral Health.

[77] Richert R., Goujat A., Venet L., Viguie G., Viennot S., Robinson P., Farges J.C., Fages M., Ducret M. (2017). Intraoral scanner technologies: A review to make a successful impression. J. Healthc. Eng..

[78] Azar B., Eckert S., Kunkela J., Ingr T., Mounajjed R. (2018). The marginal fit of lithium disilicate crowns: Press vs. CAD/CAM. Braz. Oral Res..

[79] Sason G.K., Mistry G., Tabassum R., Shetty O. (2018). A comparative evaluation of intraoral and extraoral digital impressions: An in vivo study. J. Indian Prosthodont. Soc..

[80] Hazeveld A., Slater J.J., Ren Y. (2014). Accuracy and reproducibility of dental replica models reconstructed by different rapid prototyping techniques. Am. J. Orthod. Dentofac. Orthop..

[81] Kim J.H., Kim K.B., Kim W.C., Kim J.H., Kim H.Y. (2014). Accuracy and precision of polyurethane dental arch models fabricated using a three-dimensional subtractive rapid prototyping method with an intraoral scanning technique. Korean J. Orthod..

[82] Papaspyridakos P., Chen Y.W., Alshawaf B., Kang K., Finkelman M., Chronopoulos V., Weber H.P. (2020). Digital workflow: In vitro accuracy of 3D printed casts generated from complete-arch digital implant scans. J. Prosthet. Dent..

[83] Motel C., Kirchner E., Adler W., Wichmann M., Matta R.E. (2019). Impact of Different Scan Bodies and Scan Strategies on the Accuracy of Digital Implant Impressions Assessed with an Intraoral Scanner: An In Vitro Study. J. Prosthodont..

[84] ISO (1994). ISO 5725-1: 1994, Accuracy (Trueness and Precision) of Measurement Methods and Results-Part 1: General Principles and Definitions.

[85] Zimmermann M., Ender A., Mehl A. (2019). Local accuracy of actual intraoral scanning systems for single-tooth preparations in vitro. J. Am. Dent. Assoc..

[86] Kim S.S., Jeong J.H., Lee J.I., Cho H.W. (2019). Effect of digital scans on marginal and internal discrepancies of zirconia crowns. J. Prosthet. Dent..

[87] Nedelcu R., Olsson P., Nyström I., Thor A. (2018). Finish line distinctness and accuracy in 7 intraoral scanners versus conventional impression: An in vitro descriptive comparison. BMC Oral Health.

[88] Mennito A.S., Evans Z.P., Nash J., Bocklet C., Lauer A., Bacro T., Cayouette M., Ludlow M., Renne W.G. (2019). Evaluation of the trueness and precision of complete arch digital impressions on a human maxilla using seven different intraoral digital impression systems and a laboratory scanner. J. Esthet. Restor. Dent..

[89] Mangano F.G., Admakin O., Bonacina M., Lerner H., Rutkunas V., Mangano C. (2020). Trueness of 12 intraoral scanners in the full-arch implant impression: A comparative in vitro study. BMC Oral Health.

